# Pair-EGRET: enhancing the prediction of protein–protein interaction sites through graph attention networks and protein language models

**DOI:** 10.1093/bioinformatics/btae588

**Published:** 2024-10-03

**Authors:** Ramisa Alam, Sazan Mahbub, Md Shamsuzzoha Bayzid

**Affiliations:** Department of Computer Science and Engineering, Bangladesh University of Engineering and Technology, Dhaka 1205, Bangladesh; Department of Computer Science and Engineering, Bangladesh University of Engineering and Technology, Dhaka 1205, Bangladesh; Computational Biology Department, School of Computer Science, Carnegie Mellon University, Pittsburgh, PA 15213, United States; Department of Computer Science and Engineering, Bangladesh University of Engineering and Technology, Dhaka 1205, Bangladesh

## Abstract

**Motivation:**

Proteins are responsible for most biological functions, many of which require the interaction of more than one protein molecule. However, accurately predicting protein–protein interaction (PPI) sites (the interfacial residues of a protein that interact with other protein molecules) remains a challenge. The growing demand and cost associated with the reliable identification of PPI sites using conventional experimental methods call for computational tools for automated prediction and understanding of PPIs.

**Results:**

We present Pair-EGRET, an edge-aggregated graph attention network that leverages the features extracted from pretrained transformer-like models to accurately predict PPI sites. Pair-EGRET works on a *k*-nearest neighbor graph, representing the 3D structure of a protein, and utilizes the cross-attention mechanism for accurate identification of interfacial residues of a pair of proteins. Through an extensive evaluation study using a diverse array of experimental data, evaluation metrics, and case studies on representative protein sequences, we demonstrate that Pair-EGRET can achieve remarkable performance in predicting PPI sites. Moreover, Pair-EGRET can provide interpretable insights from the learned cross-attention matrix.

**Availability and implementation:**

Pair-EGRET is freely available in open source form at the GitHub Repository https://github.com/1705004/Pair-EGRET.

## 1 Introduction

Proteins play a fundamental role in various cellular processes necessary for the survival of living organisms. Many of these processes rely on the formation of protein complexes, which involve interactions between two or more proteins. A thorough understanding of protein interfaces and the interacting residues involved is crucial in fields like disease research (Nibbe *et al.* 2011, Kuzmanov and Emili 2013), drug development (Bai *et al.* 2024, Rao *et al.* 2024), and unraveling the underlying mechanisms of basic cellular biology (Rui *et al.* 2023, Grassmann *et al.* 2024).

Protein–protein interaction sites (PPIS) are the residues of a protein that interact with other proteins’ residues to form an interface between them. There are primarily two formulations for the problem of identifying interacting residues in protein complexes. One is *partner-independent* prediction problem that tries to find the set of residues of a given isolated protein that may interact with residues of any other protein (Gainza *et al.* 2019, Mahbub and Bayzid 2022, Mou *et al.* 2023). The second form—and the one addressed in this study—is *partner-specific*, which involves the identification of interacting residues of a protein for a particular partner protein. Partner-specific methods can further be classified into those that seek to only identify which residues are part of the interface between the protein pairs (henceforth referred to as interface region prediction methods) and those that seek to predict which specific pairs of residues (one from each protein) interact with one another (henceforth referred to as pairwise PPIS prediction methods) (Fout *et al.* 2017, Sanchez-Garcia *et al.* 2019, Dai and Bailey-Kellogg 2021, Sunny *et al.* 2023). The latter is more challenging than the first and for most methods, performing the pairwise PPIS prediction task leads to the identification of interface regions. This research addresses both forms, presenting an innovative method for accurately predicting interface regions and pairwise PPIS in protein complexes.

Experimentally identifying PPIS using wet lab methods is time-consuming and costly, resulting in the rise of various computational approaches as alternatives. Computational methods like protein–protein docking models (Pierce *et al.* 2014, Yan *et al.* 2020), template-based methods (Mosca *et al.* 2013, Segura *et al.* 2016), and machine learning methods (Hou *et al.* 2017, Northey *et al.* 2018, Sanchez-Garcia *et al.* 2019, Wang *et al.* 2019) employ different techniques like computationally rotating and translating proteins to produce different poses, comparing an unknown protein with a known query protein, and training models to learn features of interacting residues, etc. However, these methods suffer from numerous shortcomings including limited coverage, inability to predict novel interactions, and challenges in feature selection.

Some recent deep learning-based methods (Fout *et al.* 2017, Liu *et al.* 2020, Mahbub and Bayzid, 2022, Wu *et al.* 2023, Lin *et al.* 2023, Bai *et al.* 2024) have demonstrated that incorporating information from both the primary amino acid sequence and the 3D structure of a protein leads to more accurate identification of PPIS. For extracting features from the primary sequence, the recently developed protein language models (Vig *et al.* 2021, Elnaggar *et al.* 2021), trained on large datasets of 1D amino acid sequences, have been proven to be effective (Mahbub and Bayzid 2022, Hasan *et al.* 2023). For encoding the 3D structural information of proteins, several geometric structures have been proposed (Gainza *et al.* 2019, Dai and Bailey-Kellogg 2021) among which graph neural networks (GNNs) (Scarselli *et al.* 2008) has proved to be useful for a number of methods (Fout *et al.* 2017, Liu *et al.* 2020, Mahbub and Bayzid 2022). GNNs have the capability to learn global and local contextual features for a residue from its neighborhood (Mahbub and Bayzid 2022) proposed an **E**dge Aggregated **GR**aph Attention N**ET**work (EGRET), a variant of GNN (Velickovic *et al.* 2018), where both node-level and edge-level features are used for calculating the attention scores allowing the model to utilize the rich structural data encoded in the edges of the graph. EGRET was proposed for the task of partner-independent prediction of interaction sites.

In this study, building on the recent successful application of transformers (Vaswani *et al.* 2017) and graph attention networks (GAT) (Velickovic *et al.* 2018) in partner-independent PPI site prediction, we propose a novel deep learning model Pair-EGRET that extends the architecture of EGRET for both pairwise PPI site and interface region predictions. Pair-EGRET integrates GNN and protein language models to effectively leverage both structural and sequence-based information. Moreover, to allow each protein to attend to the relevant features from the other protein’s residues, we adopted the concept of the cross-attention (Vaswani *et al.* 2017), widely used in natural language processing for incorporating information from multiple input sources or contexts. The combination of using transfer learning, edge-aggregated GAT networks, and cross-attention modules led to the improvement of Pair-EGRET compared to the best alternative methods on widely accepted benchmark datasets for partner-specific interaction prediction (both PPI site and interface region predictions). We have included case studies that visually inspect the predicted binding residues. We further visualized and interpreted the representations learned by Pair-EGRET. Pair-EGRET is a fully automated end-to-end pipeline, alleviating the need for time-consuming and careful hand-tuning.

## 2 Materials and methods

### 2.1 Feature representation

We represent the 3D structures of the receptor and the ligand of a complex using directed *k*-nearest neighbor graphs, denoted as $G_{\text{receptor}}$ and $G_{\text{ligand}}$, respectively. Each graph node corresponds to an amino acid residue and is connected to its *k* closest neighbors via directed edges, where *k* is a hyperparameter. The determination of nearest neighbors is based on averaging the distances between the atoms of residue pairs within a protein, which are obtained from Protein Data Bank (PDB) files (Berman *et al.* 2000). We used embedding vectors generated by ProtBERT (Vig *et al.* 2021) and some physicochemical properties of amino acids as node-level features. The distance and angle between residues served as the edge-level features for the weighted edges connecting them.

#### 2.1.1 Node-level features

Each residue *i* in a protein is associated with a feature vector $q_{i}\in R^{d_{\text{node}}}$, where $d_{\text{node}}$ represents the number of node features used in this study. We used two types of node features to represent a residue.

- Embedding-based features of the residues were extracted from the protein sequences using ProtBERT, a contextual embedding generation pipeline developed by Vig *et al.* (2021). ProtBERT captures both local and global context, including neighboring residues and overall protein structure, to generate embedding vectors $e=\{e_{1},e_{2},\ldots ,e_{N}\}$, $e_{i}\in R^{d_{\text{protbert}}}$ ($d_{\text{protbert}}=1024$ and *N* = number of residues in the given protein), which encode the structural and functional characteristics of the residues. Note that other embedding generation models available in ProtTrans (Elnaggar *et al.* 2021), such as ProtXL or ProtXLNet, can also be used instead of ProtBERT.
- Physicochemical features of amino acids were incorporated as node features. These features encompass a range of properties, including hydrophilicity, flexibility, accessibility, turns scale, exposed surface, polarity, antigenic propensity, hydrophobicity, net charge index of side chains, polarizability, solvent-accessible surface area (SASA), relative SASA, side-chain volume, and residue depth. Notably, we calculate relative hydrophobicity and polarity based on two different scales or methods, namely H11a, H12a, P11a, and P12a, respectively, to ensure a comprehensive representation of these characteristics in our analysis (Debnath and Mollah 2022). These properties of a node *i* is represented by a vector $p_{i}\in R^{d_{\text{phychem}}}$$\left(d_{\text{phychem}}=16\right)$. By concatenating the vectors $e_{i}$ and $p_{i}$ we obtained the final node features $q=\{q_{1},q_{2},\ldots ,q_{N}\}$, $q_{i}\in R^{d_{\mathrm{node}}}$ where $d_{\text{node}}=d_{\text{protbert}}+d_{\text{phychem}}=1024+16=1040$.

#### 2.1.2 Edge-level features

Similar to EGRET, the edge features of an edge from node *j* to node *i* in the graph representation of a protein is denoted by $\xi_{ji}$, where $\xi_{ji}\in R^{f_{\varepsilon }}$ and $f_{\varepsilon }$ is the number of features of the edge. We used the following two features (i.e., $f_{\varepsilon }=2$) as edge features: (i) inter-residue distance $D_{ij}$ which denotes the average distance between the atoms of the residues, and (ii) relative orientation $\theta_{ij}$ which is measured by the absolute value of the angle formed by the surface-normals of the planes passing through the alpha carbon atom ($C_{\alpha }$), the carbon atom of the Carboxyl group, and the nitrogen atom of the Amino group of each residue.

### 2.2 Architecture of Pair-EGRET

The architecture of Pair-EGRET can be discussed in two parts: (i) the architecture of EGRET, which was proposed for predicting the interaction sites of a single isolated protein and is used as the foundation of Pair-EGRET, and (ii) the proposed extension of the EGRET architecture for predicting interaction sites from pairs of proteins.

#### 2.2.1 Architecture of EGRET

In our study, we utilize the EGRET model as the foundation for our Pair-EGRET framework. The Supplementary material provides a detailed description and visualization (Supplementary Fig. S1) of the EGRET architecture.

#### 2.2.2 Extension of EGRET for pairwise prediction

In this study, we extend the EGRET architecture to identify interactions between protein pairs. Since the original EGRET was developed for single isolated proteins, we leverage its architecture for extracting useful features from the graph representations of the receptor and the ligand separately, which are subsequently analyzed using a multiheaded cross-attention layer.

**Figure 1.**
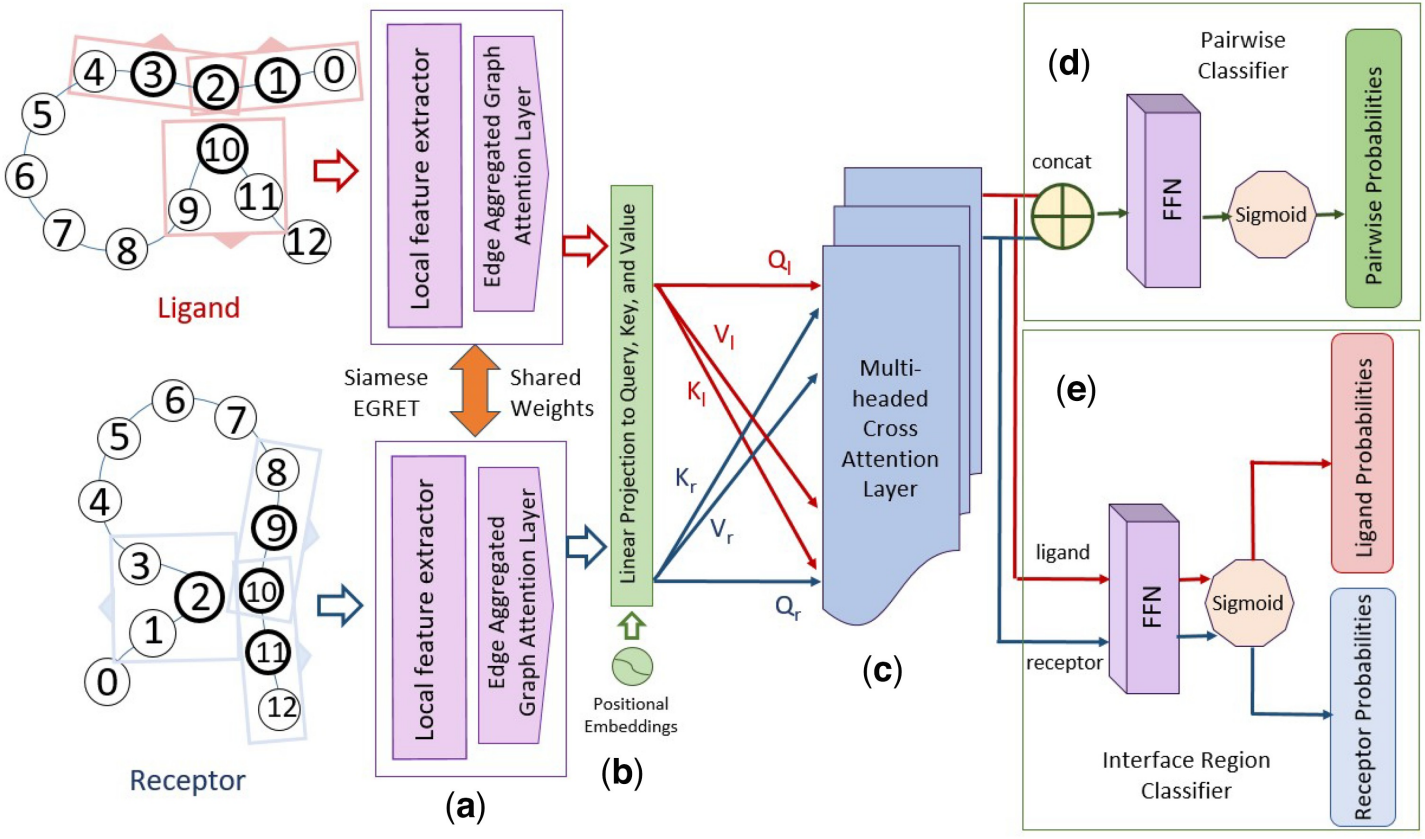
Schematic diagram of the overall pipeline of Pair-EGRET being applied to a receptor protein (r) and a ligand–protein (l). (a) Siamese EGRET network with shared weights. (b) Positional encoder being added to the individual proteins and linearly projected to three separate feature spaces—query, key, and value. (c) Both proteins transformed through the multiheaded cross-attention layer using the query vector of itself and the key and value vector of the other protein. (d) Pairwise classifier applied to the merged features of both proteins to produce pairwise probabilities of interaction. (e) Interface region classifier applied to individual proteins for producing probabilities of being part of the interface for each residue.

The core components of the Pair-EGRET architecture include (i) Siamese EGRET network, (ii) positional encoder, (iii) multiheaded cross-attention layer, (iv) pairwise classifier, and (v) interface region classifier. The first three components are connected sequentially and are common to the architecture required for both the problems we are addressing in this study, i.e., pairwise interaction site and interface region prediction. The final two layers (pairwise classifier and interface region classifier) are parallel networks that generate the outputs corresponding to these two problems. The overall end-to-end pipeline of Pair-EGRET is shown in Fig. 1, which demonstrates the major modules of Pair-EGRET being applied to a pair of proteins each containing 13 residues.

i) Siamese EGRET network

The architecture of Pair-EGRET begins with a Siamese network, which consists of a pair of identical networks containing the first two modules of EGRET (local feature extractor and edge-aggregated graph attention layer). These two networks work in tandem on the given pair of proteins (the receptor and ligand) and share their weights, enabling the Siamese network to learn a common representation of both the ligand and receptor, ensuring that the model learns a common representation of proteins and is invariant to the ordering of the partner proteins provided to it.

Given that the third module of EGRET (the node-level classifier) calculates the interaction probability for each residue independently of its partner protein in the complex, we eliminate this layer. Instead, we utilize features extracted from EGRET’s local feature extractor and graph attention (GAT) layer to encode each residue with features derived from its neighborhood, taking into account both sequential and spatial proximity.

The node features $q_{i}$ and edge features $\xi_{ij}$ corresponding to each of the graphs $G_{\text{receptor}}$ and $G_{\text{ligand}}$ are fed through the Siamese network to generate features ${\hat{h}}^{l}$ and ${\hat{h}}^{r}$, respectively. In addition to the embedding-based features used as node features in the original EGRET framework, we integrate a wide array of physicochemical features specific to each residue. Furthermore, we enhance the quality of the features generated from the Siamese network by increasing the number of convolution layers in the local feature extractor and using multiple layers of the graph attention module.

ii) Positional encoder

The positional encoder module—inspired by Vaswani *et al.* (2017)—is used in Pair-EGRET to utilize the information about the position of residues in a protein sequence. This module calculates a positional embedding for each residue and adds it to the feature representations obtained from the Siamese network. This can be useful because the location of a residue can impact its interaction with residues present in the partner protein in the complex. Following Vaswani *et al.* (2017), we defined the positional encoding for a position *pos* in the sequence as follows:

(1) $$PE(\mathrm{pos},i)=\{\begin{array}{cc} \;\text{sin}(\mathrm{pos}/10000^{\frac{i}{d_{\text{model}}}}), & \text{if}\;i\;\text{is}\;\text{even},\\ \;\text{cos}(\mathrm{pos}/10000^{\frac{i-1}{d_{\text{model}}}}), & \text{otherwise}, \end{array}$$

where $i\in [1,d_{\text{model}}]$ is the dimension of the embedding vector being calculated, and $d_{\text{model}}$ is the dimension of the input embedding vector. This function maps the position of each amino acid to a set of position-specific embeddings *PE*. *PE* is added to the feature representations ${\hat{h}}^{l}$ and ${\hat{h}}^{r}$ obtained from the Siamese network, to generate the outputs $P^{l}$ and $P^{r}$ of this layer.

$$P_{\mathrm{pos}}^{l}={\hat{h}}_{\mathrm{pos}}^{l}+PE_{\mathrm{pos}}\;,\;P_{\mathrm{pos}}^{r}={\hat{h}}_{\mathrm{pos}}^{r}+PE_{\mathrm{pos}}$$

iii) Multiheaded cross-attention layer

The multiheaded cross-attention layer employs the cross-attention mechanism to combine features originating from both receptor and ligand proteins. Through an encoder–decoder structure, this layer facilitates the mutual exchange of information between the proteins. It quantifies the influence of a ligand residue on a receptor residue and vice versa by generating attention scores that help identify relevant residue pairs within a complex. We employ multiple attention heads within this module to capture various aspects of the input features.

The cross-attention module transforms the input feature vectors into three feature spaces: query, key, and value using learnable parameters $W_{Q},W_{K}$, and $W_{V}\in R^{d_{k}\times d_{k}}$, where $d_{k}$ is a hyperparameter. Specifically, the ligand feature vector $P^{l}$ obtained from the positional encoder layer is transformed into query $Q^{l}=W_{Q}P^{l}$, key $K^{l}=W_{K}P^{l}$, and value $V^{l}=W_{V}P^{l}$, while the receptor feature vector $P^{r}$ is transformed into $Q^{r}$, $K^{r}$, and $V^{r}$ using similar equations.

To model the interaction between the proteins, the cross-attention module is applied to the ligand and receptor feature vectors separately, as defined by the following equations:

(2) $$\begin{array}{cc} \text{Attention}(Q^{l},K^{r},V^{r}) & =\text{softmax}\left(\frac{Q^{l}{\left(K^{r}\right)}^{T}}{\sqrt{d_{k}}}\right)V^{r}\\ \text{Attention}(Q^{r},K^{l},V^{l}) & =\text{softmax}\left(\frac{Q^{r}{\left(K^{l}\right)}^{T}}{\sqrt{d_{k}}}\right)V^{l} \end{array}$$

where softmax is the softmax activation function, and ${\left(\cdot \right)}^{T}$ denotes the transpose operation. To capture diverse aspects of the input features, the cross-attention module employs multiple attention heads. Each attention head is an independent attention layer that attends to different aspects of the input. Specifically, multiheaded attention is defined by the following equation:

$$\text{MultiHead}(Q,K,V)=W_{O}\text{Concat}([{\text{head}}_{1},{\text{head}}_{2},\ldots ,{\text{head}}_{N_{h}}]),$$

where ${\text{head}}_{i}=\text{Attention}(Q,K,V)$ is the output of the *i*-th attention head, $N_{h}$ is the number of total attention heads, and $W_{O}$ is a learnable parameter used to project the concatenated attention heads back to the original feature space.

The outputs of the multiheaded attention are then added to the input of the module through a residual connection (He *et al.* 2016), which enables the model to capture both the attended features and original features, followed by layer normalization (Ba *et al.* 2016), which stabilizes the model. The final outputs $M^{l}$ and $M^{r}$ are defined by the following equations:

$$\begin{array}{c} M^{l}=\text{LayerNorm}(P^{l}+\text{MultiHead}(Q^{l},K^{r},V^{r}))\\ M^{r}=\text{LayerNorm}(P^{r}+\text{MultiHead}(Q^{r},K^{l},V^{l})) \end{array}$$

iv) Pairwise classifier

The pairwise classifier layer in Pair-EGRET produces the final output for our first task: Pairwise PPIS prediction. For each residue pair $\left(l_{i},r_{i}\right)$, this module generates an output $O_{i}\in \{0,1\}$ where $O_{i}=1$ would indicate that the residue $l_{i}$ in the ligand and the residue $r_{i}$ in the receptor are an interacting pair, whereas $O_{i}=0$ would indicate otherwise.

For each residue pair, we extract the node features $M_{l_{i}}^{l}$ and $M_{r_{i}}^{r}$ from the outputs $M^{l}$ and $M^{r}$ generated by the cross-attention module and concatenate them. This representation of the residue pair is passed through a feed-forward network (FFN) that incorporates a combination of fully connected layers, nonlinear activation functions such as ReLU and Leaky ReLU, and finally, a Sigmoid activation function to predict the probability of interaction between the nodes $l_{i}$ and $r_{i}$.

Similar to Fout *et al.* (2017), to ensure invariance to the ordering of the receptor and ligand, we concatenate the features in both possible orders, resulting in two predictions:

$$O_{i}^{rl}=\sigma (\text{FFN}(M^{r}r_{i}\|M^{l}l_{i})),\;O_{i}^{lr}=\sigma (\text{FFN}(M^{l}l_{i}\|M^{r}r_{i}))$$

where FFN is the feed-forward network and σ is the Sigmoid activation function. Finally, we take the average of these two predictions to generate the final output probability $O_{i}$ for the residue pair $\left(l_{i},r_{i}\right)$:

$$O_{i}=\frac{1}{2}(O_{i}^{rl}+O_{i}^{lr})$$

v) Interface region classifier

The interface region classifier generates the final outputs for our second task: identifying the interface region of protein complexes. For each residue $l_{i}$ of the ligand or $r_{j}$ of the receptor, this module generates an output $O_{i}^{l}$ or $O_{j}^{r}$ which indicates whether the residues are a part of the interface of the corresponding complex or not.

In this layer, instead of concatenating the outputs $M^{l}$ and $M^{r}$ generated by the cross-attention layer, we pass $M^{l}$ and $M^{r}$ individually through an FFN. Similar to the pairwise classifier, this network incorporates fully connected layers and activation functions that linearly transform the feature vectors and finally a Sigmoid activation function generates the probabilities of each residue being part of the interface region:

$$O_{i}^{l}=\sigma (\text{FFN}(M^{l}l_{i})),\;O_{j}^{r}=\sigma (\text{FFN}(M^{r}r_{j}))$$

The algorithmic details of the EGRET module and Pair-EGRET are presented in Algorithm 1 and Algorithm 2 in the Supplementary material.

### 2.3 Datasets

We evaluated Pair-EGRET on three benchmark datasets: (i) Docking Benchmark version 5.0 (DBD5) (Vreven *et al.* 2015), (ii) Dockground X-ray unbound benchmark (Kundrotas *et al.* 2018), and (iii) A subset of the MaSIF (Gainza *et al.* 2019) dataset. Detailed descriptions of these datasets are included in the Supplementary material. For each complex, the DBD5 and Dockground datasets contain the atomic coordinates of the amino acid residues of the proteins in both their bound and unbound forms. We used the unbound structures to produce the input features for the model and the bound structures to identify interacting residues in protein pairs. Consistent with prior work (Afsar Minhas *et al.* 2014, Fout *et al.* 2017, Sanchez-Garcia *et al.* 2019), we defined two residues to be interacting if any of their non-Hydrogen atoms were within 6 Å of one another. The MaSIF dataset lacks the atomic coordinates of the proteins in their unbound form. So we used the bound structures for producing the input features for the model while evaluating the MaSIF dataset.

We used the DBD5 and Dockground datasets for evaluating Pair-EGRET and other contemporary methods on the task of pairwise PPIS prediction. DBD5 was also used along with the MaSIF dataset for the interface region prediction task. The choice of datasets in each case was made based on the availability of evaluation scores of existing methods on the intended task so that a fair and comprehensive analysis of performance could be presented.

In all of these datasets, the number of positive samples (interacting residue pairs) is very small compared to the number of negative samples (non-interacting residue pairs) with the ratio of positive-to-negative samples being close to almost 1:1000. To address this imbalance, similar to Fout *et al.* (2017) and Liu *et al.* (2020), during training, we downsampled the negative samples to obtain a 1:10 ratio of positive-to-negative samples. For validation and testing, we preserved the original ratio of the samples present in the datasets. The summary of the dataset sizes and data partitions is presented in Supplementary Table S1.

### 2.4 Methods compared

The supplementary material provides brief summaries of the baseline methods for protein–protein interaction site and interface region prediction that were used for comparison in this study.

### 2.5 Performance evaluation

As the datasets we are using are highly imbalanced, accuracy is not a meaningful evaluation metric for either of the tasks. We used AUROC (area under receiver operating characteristic curve) and AUPRC (area under precision-recall curve) as our primary evaluation metrics. AUROC and AUPRC are threshold independent and therefore, are appropriate measures of performance for evaluating performance on high-imbalance datasets (Li *et al.* 2021). We calculated the AUROC for each of the test complexes and compared the performance of the methods using the median AUROC scores. This reduces the probability of extreme change in the performance resulting from very small or very large complexes (Liu *et al.* 2020). We also compared the performance of Pair-EGRET in terms of AUPRC, even though the extreme class imbalance resulted in poor AUPRC scores for all the methods in the pairwise interaction site prediction task.

The value of the same evaluation metric (e.g., AUROC, AUPRC) can differ between pairwise interaction site prediction and interface region prediction, even for the same complex. This difference stems from how true positives (TP), false positives (FP), true negatives (TN), and false negatives (FN) are defined for each task. For pairwise PPIS prediction, these metrics are computed based on specific residue-residue interactions, with a TP occurring when the model correctly identifies an interaction between a particular residue from one protein and a specific residue from another. In contrast, for interface region prediction, the metrics are based on whether a residue belongs to the interaction interface. Here, a TP occurs when a residue is correctly predicted to be part of the interface (i.e., interacting with at least one residue on the other protein), without focusing on specific residue pairs. The definitions of FP, TN, and FN also similarly differ between these tasks, leading to variations in the resulting evaluation metric values.

### 2.6 Precision analysis

To address the impact of the datasets’ imbalance on the precision scores of Pair-EGRET, we have included some precision analysis results. For the interface region prediction task, precision vs. threshold and precision vs. recall curves are added to better understand the precision scores. For the task of pairwise PPIS prediction, we evaluated the precision of Pair-EGRET on the top *N* (N∈10,20,…,100) high-confidence predictions per complex. These predictions represent residue pairs identified by Pair-EGRET as interaction sites with very high probability scores.

### 2.7 Working parameters for Pair-EGRET

We performed a hyperparameter tuning process to optimize the performance of Pair-EGRET on both pairwise PPIS prediction and interface region prediction tasks. Supplementary Table S2 presents the values of the parameters that we used across all the datasets analyzed in this study.

## 3 Results and discussions

We conducted a comprehensive evaluation study to compare the performance of Pair-EGRET, a novel method proposed in this study, with state-of-the-art conventional machine learning methods, convolutional neural network (CNN)-based methods, and GNN-based methods, using widely accepted benchmark datasets for pairwise interaction site and interface region prediction.

### 3.1 Pairwise PPIS prediction results

#### 3.1.1 Results on DBD5

For the task of pairwise interaction site prediction, we compared the median AUROC and AUPRC scores of Pair-EGRET with the state-of-the-art machine learning, CNN, and GNN-based methods (Duvenaud *et al.* 2015, Atwood and Towsley 2016, Fout *et al.* 2017, Schütt *et al.* 2017, Sanchez-Garcia *et al.* 2019, Townshend *et al.* 2019, Satorras *et al.* 2021, Jing *et al.* 2021). It is evident from Table 1 that Pair-EGRET outperforms all the other methods in terms of our primary evaluation metric, median AUROC with a score of 0.88828. We note that all the methods achieved low AUPRC scores, indicating the complexity of the task and the imbalanced nature of the dataset. However, our method achieves an AUPRC score of 0.0173 which is comparable to the best-performing method DCNN (Atwood and Towsley 2016) with an AUPRC of 0.018.

**Table 1.** Comparison between the predictive performance of different methods in predicting pairwise PPIS of the test complexes of DBD5 and Dockground.^a^

DBD5						Dockground	
Method	Median AUROC	AUPRC	Method	Median AUROC	AUPRC	Method	Median AUROC
BIPSPI	0.878	–	DTNN	0.867	0.007	BIPSPI+	0.831
SASNet	0.876	–	NEA	0.876	0.012	Pair-EGRET	**0.8747**
DCNN	0.828	**0.018**	EGNN	0.829	–		
NGF	0.865	0.007	GVP-GNN	0.885	–		
Pair-EGRET	**0.888**	0.0173					

a Scores for the baseline methods on DBD5 are reported from Fout *et al.* (2017), Liu *et al.* (2020), and Wu *et al.* (2023). The best results are shown in bold. Values not reported by corresponding studies are indicated by ‘–’.

#### 3.1.2 Results on Dockground

Although the unbound benchmark version 4 of Dockground is less explored for the pairwise PPIS prediction task, we evaluated Pair-EGRET on this benchmark because of the varying levels of difficulty it offers making it more diverse than DBD5. From Table 1, we can see that compared to BIPSPI+ (Sanchez-Garcia *et al.* 2022)—one of the few methods evaluated on Dockground for this task—Pair-EGRET performs substantially better with a median AUROC of 0.8747, highlighting its robustness in identifying interaction sites in relatively difficult complexes.

To ensure that structural similarity between train and test proteins does not lead to overestimation of the model’s performance, we created subsets of the test dataset of DBD5 by removing complexes from the test set that have structural similarity with at least one complex in the training set. The structural similarity between complexes was determined based on RMSD (root mean square deviation) values calculated using the combinatorial extension algorithm (Shindyalov and Bourne 2001). Lower RMSD values between complexes indicate higher structural similarity, while higher RMSD indicates less such similarity. In Supplementary Table S3, we have shown the performance of Pair-EGRET on different subsets of the DBD5 test set created by removing complexes that have RMSD scores below different thresholds. The results suggest that structural similarity with the training set has no notable impact, indicating that the predictive performance of individual complexes in the test set is fairly independent of their structural similarity to the training set.

### 3.2 Interface region prediction results

#### 3.2.1 Results on DBD5

We compared Pair-EGRET with BIPSPI, BIPSPI+, and PInet (Dai and Bailey-Kellogg 2021) for interface region prediction on the DBD5 test dataset. In addition to AUROC and AUPRC, we also report the precision and recall scores of the methods in Table 2 for a fair comparison and consistency with the other prior studies that analyzed this dataset. Pair-EGRET substantially outperforms all the other methods under two evaluation metrics—AUROC and recall. Remarkably, the AUROC score of Pair-EGRET is 0.924 which is 8.96% higher than the second best method BIPSPI+. However, Pair-EGRET performed worse than other methods in terms of precisions and AUPRC on this particular dataset.

**Table 2.** Performance evaluation of different methods in identifying interface regions of test complexes of DBD5 and MaSIF.^a^

DBD5					MaSIF		
Method	AUROC	AUPRC	Precision	Recall	Method	AUROC	AUPRC
BIPSPI	0.822	0.410	0.391	0.558	SPPIDER	0.65	–
					MaSIF geom*	0.68	–
BIPSPI+	0.848	0.4653	0.438	0.573	MaSIF	0.87	–
					PInet geom*	0.75	0.30
PInet	0.753	**0.596**	**0.492**	0.723	PInet	0.88	0.45
Pair-EGRET	**0.924**	0.275	0.255	**0.746**	Pair-EGRET	**0.9583**	**0.5938**

a geom* indicates models that only use geometric features of proteins. The best results are shown in bold. Values not reported in the MaSIF study (Dai and Bailey-Kellogg 2021) are indicated by ‘–’.

#### 3.2.2 Results on MaSIF

Our assessment of Pair-EGRET on the MaSIF dataset involved a comparison with MaSIF (Gainza *et al.* 2019), SPPIDER (Porollo and Meller 2007), and PInet. Pair-EGRET significantly outperformed all other methods in terms of both AUROC and AUPRC. Pair-EGRET achieved an AUROC score of 0.9583, which is 8.89% higher than the next best method PInet. Additionally, Pair-EGRET achieved a much higher AUPRC score (0.5938) which is around 15% higher than PInet. Since the MaSIF benchmark is substantially large and contains a collection of diverse and uncurated protein complexes (Dai and Bailey-Kellogg 2021), the substantially higher AUROC and AUPRC scores of Pair-EGRET than other methods are indicative of the model’s ability to learn a better generalized representation of interface residues of ligand and receptor proteins compared to other methods.

### 3.3 Analysis of precision scores of Pair-EGRET

Supplementary Fig. S2 presents the precision versus classification threshold plots and precision-recall curves for the interface region prediction task on both the DBD5 and MaSIF datasets. This figure clearly shows higher precision values at higher thresholds for both datasets and reasonably large areas under the precision-recall curves.

Supplementary Table S4 shows the precision of Pair-EGRET on the top *N* (N∈10,20,…,100) high-confidence predictions per complex on the task of pairwise PPIS prediction for both the DBD5 and Dockground test sets. As shown in the table, the precision scores for these high-confidence residue pairs are notably high for both datasets.

### 3.4 Performance evaluation on antibody–antigen complexes

One of the most significant applications of PPI site prediction is in the study of host–pathogen interactions, which involve the interaction between a host organism (e.g., human, animal, or plant) and a pathogen (e.g., bacterium, virus, fungus, or parasite) that causes disease via antibody–antigen interactions (MacCallum *et al.* 1996). This field has traditionally been challenging due to the highly flexible structures of antibodies and antigens (MacCallum *et al.* 1996, Jin *et al.* 2022). To evaluate the performance of Pair-EGRET on antibody–antigen complexes, we extracted 17 such complexes from the test set of DBD5 and assessed our model. The results are presented in Supplementary Table S5.

For comparison, we also evaluated AlphaFold-Multimer (AFM) (Evans *et al.* 2022) on these complexes. AFM, specialized in predicting protein complexes, has been rigorously trained on all protein complexes in the PDB up to 30 April 2018, with the current variant further fine-tuned on complexes until 30 September 2021 (https://github.com/google-deepmind/alphafold/blob/main/docs/technical_note_v2.3.0.md). All the antibody–antigen complexes in our subset were deposited and released before 2018, thus falling within the training set of AFM. This creates an inherent bias in the comparison between AFM and Pair-EGRET, whose training set is strictly non-overlapping with this antibody–antigen test set. Nonetheless, Pair-EGRET demonstrates remarkable performance, surpassing AFM in several cases. Specifically, among the 17 complexes, Pair-EGRET achieves higher AUROC in 16 instances, higher AUPRC in 5, higher F1-score in 6, higher precision in 6, and higher recall in 12 instances. Despite the inherent bias in this comparison, the superior performance of Pair-EGRET over AFM on several complexes within AFM’s training set underscores the potential of Pair-EGRET in studying host–pathogen interactions.

### 3.5 Interpretability analysis: patterns of cross-attention scores

**Figure 2.**
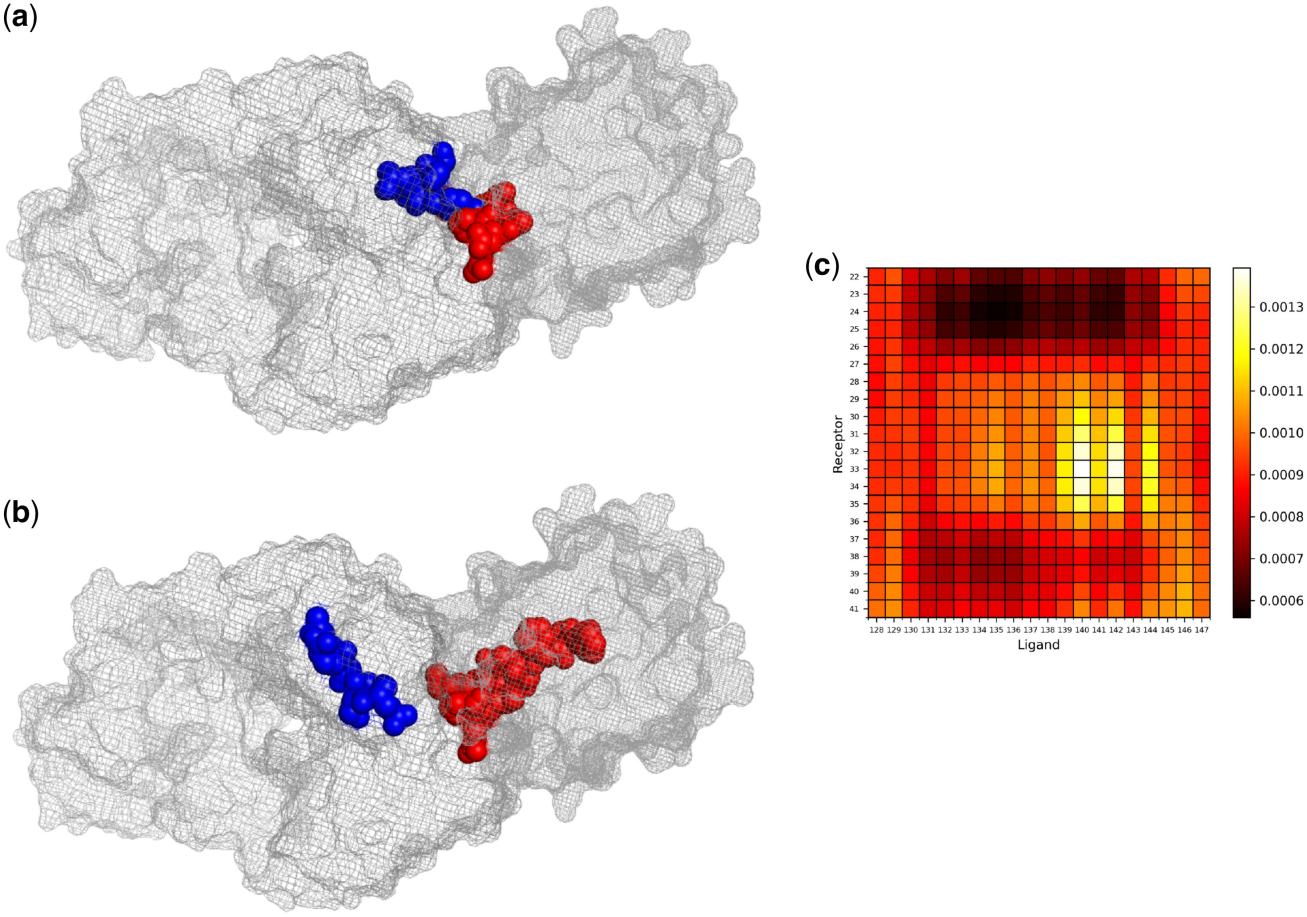
Patterns of cross-attention scores in a 20 residue window of chain A of the ligand and chain L of the receptor of a representative complex (PDB ID 3HI6). (a) PyMOL visualization of the residues corresponding to the lighter regions (high attention scores) of the heatmap. (b) PyMOL visualization of the residues corresponding to the darker regions (low attention scores) of the heatmap. (c) Heatmap of the attention scores generated by the multiheaded cross-attention layer of Pair-EGRET.

One of the noteworthy contributions of this study is introducing the cross-attention mechanism for pairwise protein interaction site and interface region prediction. We investigated the behavior of the multiheaded cross-attention layer of Pair-EGRET to provide insights into how this layer learns the correlation between residues from two different proteins. Figure 2c shows the heatmap representation of the attention scores generated for a representative protein (PDB ID 3HI6) from the DBD5 test dataset. For the convenience of visualization, we show a small window of the attention score matrix corresponding to 20 residues (residue IDs 128–147) from chain A of the ligand and 20 residues (residue IDs 22–41) from chain L of the receptor.

Interestingly, the residue pairs that were given the highest attention scores (lighter color in the heatmap) either correspond to interacting residue pairs (according to the dataset labels) or are very close to such interacting pairs in the Euclidean space. As shown in the PyMOL (DeLano 2002) visualization of Fig. 2a, the residues having relatively high attention scores are very close in space and likely belong to the interface region of the protein complex. On the other hand, pairs with lower attention scores (darker color in the heatmap) correspond to the residues shown in Fig. 2b. These residue pairs are visibly apart from each other and some are even inside the surface of the proteins. These results demonstrate the meaningful relationship between attention scores generated by the cross-attention layer of Pair-EGRET and the characteristics of interacting residue pairs in a complex.

### 3.6 Case study

**Figure 3.**
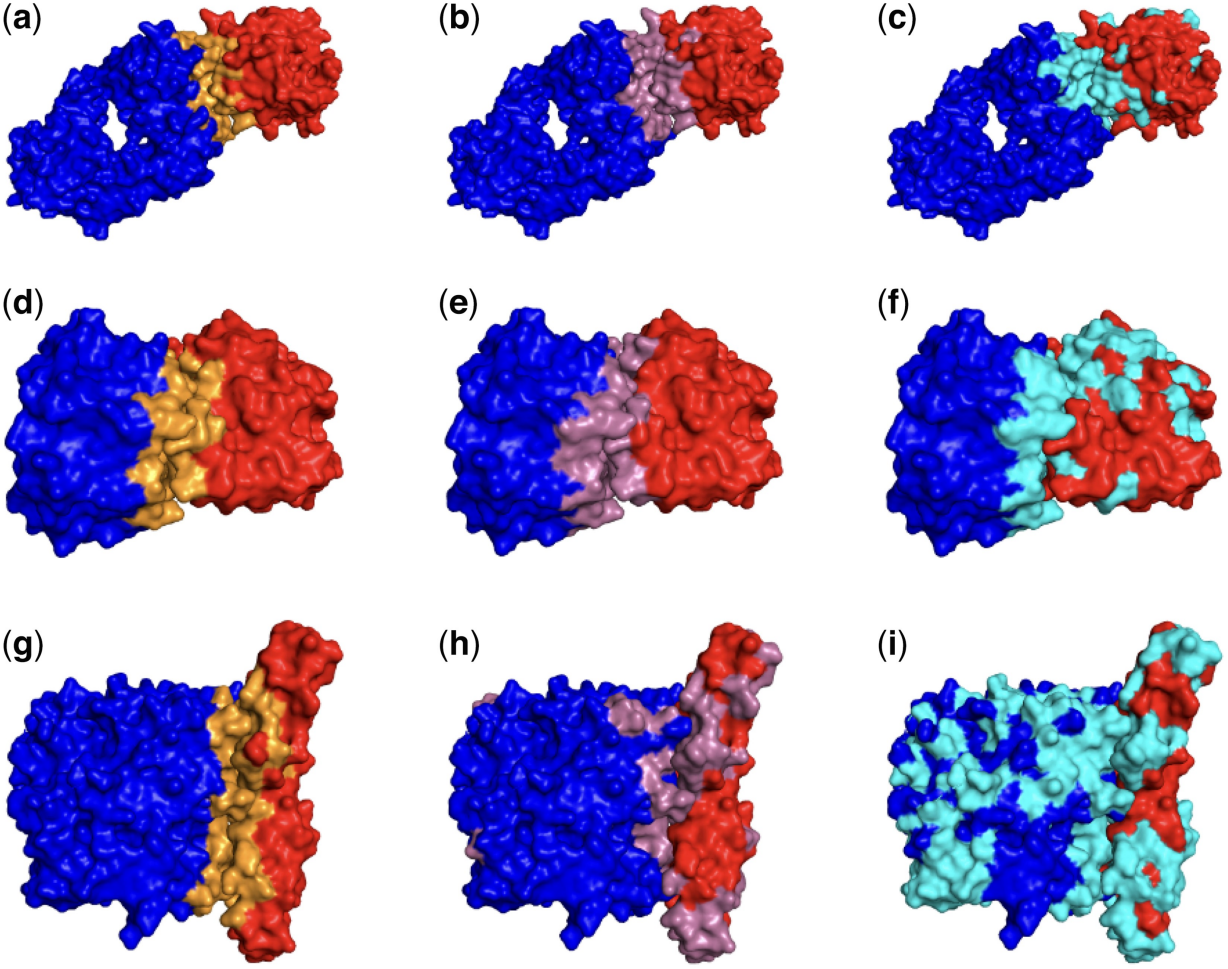
PyMOL visualization of three representative complexes (PDB ID 3HI6, 1JTD, and 3L89) from the test set of DBD5 in bound form. The right and left surfaces on each subfigure represent the ligand and receptor proteins respectively. The true (a), predicted by Pair-EGRET (b), and predicted by NEA (c) interface regions of 3HI6. The true (d), predicted by Pair-EGRET (e), and predicted by NEA (f) interface regions of 1JTD. The true (g), predicted by Pair-EGRET (h), and predicted by NEA (i) interface regions of 3L89.

In Fig. 3, we visualized three representative complexes (PDB ID 3HI6, 1JTD, and 3L89) from the DBD5 test dataset using the PyMOL software to perform a qualitative analysis of Pair-EGRET’s performance in identifying interface regions. We also visualized the interface regions predicted by NEA (pairwise PPISP prediction method developed by Fout *et al.* 2017) to compare our results with a competitive method. Most of the existing methods are not publicly available (either as user-friendly software packages or web servers). Consequently, we were unable to include additional methods in this visual inspection. Supplementary Table S6 presents the values of precision, true positive rate, and false positive rate of these three complexes for both models.

For the first two complexes (PDB ID 3HI6 and 1JTD), we visualized the interface residues identified by the interface region classifier of Pair-EGRET with very high (95%–99%) probability. In the case of NEA, the overall probability scores produced by this model were lower compared to Pair-EGRET, with almost no residues with scores over 90%. So we presented the residues that were predicted by NEA to have greater than 80% probability of interacting with at least one residue of the partner protein. For the complex with PDB ID 3L89, lower thresholds of probability scores (80% for Pair-EGRET and 65% for NEA) were used for visualizing interface regions as the overall probability scores generated by both models were low for this complex.

It is evident from the figure that compared to the predictions generated by NEA (Fig. 3c, f, i), the interface residues identified by Pair-EGRET (Fig. 3b, e, h) are less scattered and more concentrated toward the true interface regions (Fig. 3a, d, g). Moreover, predictions from Pair-EGRET accurately encompass the entirety of the actual interface whereas NEA misses parts of the interface regions in both complexes. Furthermore, Pair-EGRET produced notably lower numbers of false negative predictions than NEA. The complex with PDB ID 3L89 was selected from among the worst-performing cases for Pair-EGRET. Despite this, Pair-EGRET generates substantially fewer FN than NEA and its positive predictions are more concentrated in the true positive regions.

### 3.7 Analysis of model performance

We analyzed the impact of different features and modules of Pair-EGRET on its performance. These results are presented in Section 1.5 and Supplementary Tables S7–S9.

## 4 Conclusions

We presented Pair-EGRET, a novel deep learning method for accurately identifying pairwise interaction sites and interface regions of protein complexes. We have demonstrated the effectiveness of using an edge-aggregated GAT as well as the cross-attention mechanism in the context of pairwise interaction prediction. Our systematic analyses of the performance of different methods under various model conditions indicate the predictive power and effectiveness of Pair-EGRET in pairwise PPI site and interface region predictions. Furthermore, Pair-EGRET offers a more interpretable framework than the typical black-box deep neural network methods. This study can be extended in several directions. We utilized the protein language model ProtTrans for feature generation. A more recent and larger language model (ESM-2) (Lin *et al.* 2022) for proteins, released by Facebook Research, can be utilized for producing better predictions by Pair-EGRET. The increasing availability of structure-known proteins has led to the assembly of several larger datasets (Townshend *et al.* 2019, Gainza *et al.* 2019) that can be used for further training our model leading to enhanced performance.

## Supplementary Material

btae588_Supplementary_Data

## Data Availability

The datasets underlying this article were derived from sources in the public domain. The preprocessed versions of the datasets used in this work are available at https://zenodo.org/records/10215073.
