# Supplementary material for: Pair-EGRET: enhancing the prediction of protein–protein interaction sites through graph attention networks and protein language models
Source: Bioinformatics. 2024 Oct 3;40(10):btae588. doi: 10.1093/bioinformatics/btae588 (PMC11495673; doi:10.1093/bioinformatics/btae588)
Supplement: btae588_Supplementary_Data [file btae588_supplementary_data.pdf]

Supplementary Materials to Pair-EGRET: enhancing the prediction of protein-protein interaction sites through graph attention networks and protein language models

Ramisa Alam[1], Sazan Mahbub[1,2], and Md. Shamsuzzoha Bayzid[1,*]

[1]Department of Computer Science and Engineering
Bangladesh University of Engineering and Technology
Dhaka-1205, Bangladesh
[2]Computational Biology Department, School of Computer Science
Carnegie Mellon University, Pittsburgh, PA 15213, USA
[*]To whom correspondence should be addressed.

These supplementary materials present additional details about the proposed methods, model parameters, and results on antibody-antigen interactions.

1 Supplementary Text

1.1 Architecture of EGRET

We discuss the structures and functionalities of the three core components of the EGRET model to make this paper self-contained and comprehensive.

Local feature extractor

The local feature extractor captures local interactions of the protein residues with other "sequentially closer" residues (not necessarily close in Euclidean space) while reducing the dimensionality of the node-level features. A one-dimensional convolutional neural network with a small odd number window size is used to encode the node feature vectors $q = \{q_1, q_2, ..., q_N\}$ into a new condensed and neighbor-aware feature representation $h = \{h_1, h_2, ..., h_N\}$, $h_i \in \mathbb{R}^{f_n}$, where $f_n < d_{protbert}$.

Edge-aggregated graph attention layer

The edge-aggregated graph attention layer transforms the features h_i of the node i by encoding the three-dimensional structural information of its neighborhood N_i. This layer uses a modified version of the original graph attention layer [Velickovic et al., 2018] and aggregation process used in various GNN-based architectures [Kipf and Welling, 2017, Velickovic et al., 2018]. In the original aggregation process, the node features are transformed by taking a weighted average of the neighborhood node features using the equation: $\hat{h_i} = \sigma(\sum_{j \in N_i} \gamma_{ij} W^v h_j)$ where, $W^v \in \mathbb{R}^{f_n \times f_n}$ is a learnable parameter and γ_{ij} is the attention score calculated from h_i and h_j that represents the importance of the features of node j to node i. EGRET improves upon this method by incorporating edge features during the calculation of attention scores and the aggregation process, resulting in a new scoring function e_{ji} and attention distribution α_{ji}. These metrics are obtained from the following equations.

$$e_{ji} = \Omega(W^\alpha [W^v h_i || W^v h_j || W^p \xi_{ji}]) \tag{1}$$

$$\alpha_{ji} = softmax(e_{ji}) = \frac{exp(e_{ji})}{\sum_{k \in N_i} exp(e_{ki})} \tag{2}$$

Finally, the node and edge features are aggregated using the equation

$$\hat{h_i} = \sigma(\sum_{j \in N_i} \alpha_{ji} W^v h_j + \sum_{j \in N_i} \alpha_{ji} W^\epsilon \xi_{ji}) \, || \, h_i \tag{3}$$

Here, $W^\alpha \in \mathbb{R}^{2f_n + f_e}$, $W^v \in \mathbb{R}^{f_n \times f_n}$, $W^p \in \mathbb{R}^{f_e \times f_e}$ and $W^\epsilon \in \mathbb{R}^{f_n \times f_e}$ are learnable parameters, $||$ is the concatenation operator, and $\Omega(.)$ and $\sigma(.)$ are activation functions.

Node level classifier

This final layer linearly transforms the aggregated features obtained from the previous layer $\hat{h_i}$ and applies sigmoid activation to generate interaction probabilities for each residue of a sequence.

Figure S1 shows the overall end-to-end pipeline of EGRET. It demonstrates EGRET being applied to a dummy protein with 13 residues.

1.2 Summary of Datasets

i) Docking Benchmark version 5.0 (DBD5) is widely recognized as the standard benchmark for evaluating pairwise PPIS prediction and interface region identification. The dataset includes structures of 230 complexes from the protein data bank (PDB) [Berman et al., 2000] with amino acid sequence lengths of the constituent proteins varying from 29 to 2128. Training and validation on DBD5 were performed using the 175 complexes present in version 4.0 of Docking Benchmark (DBD4) [Hwang et al., 2010]. We performed an 80%-20% partition of the 175 complexes stratifying them by the difficulty provided in [Vreven et al., 2015]. For testing, we used a set of 55 complexes that were added in the update from DBD4 to DBD5. This time-based split of the dataset simulates the ability of the model to predict unreleased complexes, as opposed to a random split which has more training/testing cross-contamination. [Townshend et al., 2019]

ii) Dockground is another benchmark used for evaluating pairwise interaction site prediction models. The dataset contains a diverse array of protein complexes of varying difficulties. Compared to DBD5, it has fewer proteins with rigid bodies and more with higher difficulty levels. In our experiments, we used the unbound docking benchmark set 4 of the Dockground dataset containing 396 complexes with only 77 complexes shared with DBD5. We used 236 complexes for training, 60 complexes for validation, and 100 complexes for testing Pair-EGRET on the task of pairwise interaction site prediction.

(iii) MaSIF is a relatively large dataset containing a total of 3362 complexes taken from the PRISM [Ogmen et al., 2005] list of nonredundant proteins, the ZDock benchmark [Pierce et al., 2014], PDBBind [Wang et al., 2005], and SabDab [Dunbar et al., 2014] dataset. We used the curated subset of MaSIF used by PInet [Dai and Bailey-Kellogg, 2021] which excludes complexes with interface regions smaller than 1% of the size of the ligand. We also excluded complexes with receptor or ligand sequence lengths smaller than the minimum neighborhood size required by the edge-aggregated graph attention layer of EGRET. This resulted in a dataset containing 3147 complexes, which was split into 1890 training, 470 validation and, 787 test complexes. We used this subset of MaSIF for evaluating Pair-EGRET in identifying interface regions of complexes. This benchmark uses bound conformations of proteins to produce features for training the models. Consistent with other methods, only for this benchmark, we used the provided bound conformations for generating the node and edge-level features of Pair-EGRET.

1.3 Summary of Methods Compared

BIPSPI [Sanchez-Garcia et al., 2019] is a machine learning-based method that uses XGBoost classifiers [Chen and Guestrin, 2016] and a novel scoring function to predict pairwise interaction sites from sequence-based and structural features obtained from proteins present in a complex.

SASNet [Townshend et al., 2019] is a three-dimensional convolutional neural network (CNN) based model that uses spatial coordinates and identities of atoms present in residues as input features to predict pairwise PPIS.

DCNN [Atwood and Towsley, 2016] is a neural network model for graph-structured

protein data that applies "diffusion convolution" operations to learn node features from neighborhoods and applies dense layers to predict pairwise interactions.

NGF [Duvenaud et al., 2015] is a graph convolutional neural network that operates on the molecular graph representation of proteins. It aggregates node and edge features, applies a linear transformation to the node features, and performs a non-linear transformation to classify interacting residue pairs.

DTNN [Schütt et al., 2017] is a graph neural network (GNN) based model that uses the representation of atoms as vectors, models the non-linear coupling between atomic features and inter-atomic distances through low-rank tensor factorization, and identifies pairwise interaction sites by capturing pairwise atomic interactions.

NEA [Fout et al., 2017] is another GNN-based method that performs aggregation and linear transformation techniques on both node and edge features followed by residual connections and non-linear transformations of node features to identify pairwise interaction sites.

EGNN [Satorras et al., 2021] introduces a GNN model that is equivariant to rotations, translations, reflections, and permutation of graph representations of proteins and predicts interactions by predicting molecular properties of proteins.

GVP-GNN [Jing et al., 2021] introduces geometric vector perceptrons (GVPs), which combine the geometric and relational properties of protein structures by incorporating both scalar and vector features in a graph neural network (GNN), enabling tasks such as protein interaction site prediction.

BIPSPI+ [Sanchez-Garcia et al., 2022] is an improved version of BIPSPI, with enhanced predictive performance due to training on datasets tailored to specific types of protein interactions and interfaces (e.g., homo/hetero).

PInet [Dai and Bailey-Kellogg, 2021] is a geometric deep learning framework based on PoinNet [Qi et al., 2017] which represents protein structures as point clouds encoding geometric and physicochemical properties and identifies protein interface regions by learning features capturing surface complementarity.

SPPIDER [Porollo and Meller, 2007] uses several machine learning techniques, including LDA, SVM, and neural networks for protein interface region prediction by integrating relevant solvent accessibility (RSA) with high-resolution structural data of proteins.

MaSIF [Gainza et al., 2019] uses a molecular surface representation of proteins, extracting overlapping radial patches with geometric and chemical features via a soft polar grid and geodesic convolution for identifying interface regions of proteins.

1.4 Analysis of model performance

We analyzed the impact of different features and modules of Pair-EGRET on its performance.

1.4.1 Impact of different node-level features

Supplementary Table S7 shows the impact of different node-level features on the median AUROC scores of Pair-EGRET in predicting pairwise PPIS from DBD5 test complexes. The results highlight that adding ProtBERT-based and physicochemical features improves the median AUROC score of Pair-EGRET by 10.168% and 8.518% respectively,

indicating that the model may be benefiting from the patterns captured by ProtBERT embeddings and the physical characteristics represented by the physicochemical features.

1.4.2 Impact of different modules of Pair-EGRET

In Supplementary Table S8, we analyzed the impact of different core modules of Pair-EGRET, particularly the positional encoder and the multi-headed cross-attention layer on its performance. The addition of the positional encoder introduces a 3.837% improvement in the median AUROC of Pair-EGRET for pairwise PPIS prediction in DBD5 complexes, while the cross-attention module introduces an improvement of 2.508%. This strengthens our argument that the positional encoder enhances sequential context, and the cross-attention module enables residues to access relevant information from the residues of the partner protein.

We also conducted additional ablation studies to assess the performance of models featuring simpler architectures when equipped with the enhanced physicochemical and ProtBERT-based features of Pair-EGRET. Specifically, we substituted the first three modules of Pair-EGRET with less complex architecture and presented the pairwise PPIS prediction results for DBD5 test complexes using these modified models (see Supplementary Table S9). The architectures we considered for this analysis include a Siamese feed-forward network solely composed of fully connected layers, a CNN-based network employing 1D convolution operations on protein sequences, an attention-based model integrating a positional encoder and a cross-attention module, and a graph attention network [Velickovic et al., 2018] with a single graph convolution layer [Kipf and Welling, 2017]. The results in the table reveal that the enhanced features used in this study yield reasonably good results even for very simple architectures such as Siamese FFN and 1D CNN. However, the incorporation of GAT networks or attention modules significantly improves the model's performance. Notably, the GAT network with a single convolution layer achieves the highest median AUROC score among these models. These findings suggest that the performance boost in Pair-EGRET can be attributed to both the enhanced features utilized in this study and the effective architecture of Pair-EGRET. The model not only relies on improved features but also excels in accurately capturing the contextual intricacies conveyed by these features, ultimately contributing to a significant overall performance improvement.

1.5 Algorithmic details of Pair-EGRET

Algorithm 1 EGRET Architecture

```
Function: LocalFeatureExtractor(node_features, window_size)
  condensed_features ← 1DConvolution(node_features, window_size)
  return condensed_features

Function: EdgeAggregatedAttention(node_features, edge_features, neighbors)
  Initialize weights
   for i ∈ range(len(node_features)) do
      for j ∈ neighbors[i] do
         attention_scores_ij ← weighted concatenation(node_features[i],
               node_features[j], edge_features[i][j])
         attention_scores_ij ← softmax(attention_scores_ij)
      end for
   end for
   for i ∈ range(node_features) do
      aggregation ← 0
      for j ∈ neighbors[i] do
         aggregation ← aggregation +
           attention_scores_ij · weighted sum(node_features[j], edge_features[j])
      end for
      aggregated_features[i] ← concatenate (sigmoid(aggregation, node_features[i]))
   end for
  return aggregated_features

Function: NodeLevelClassifier(aggregated_features)
  Initialize classifier parameters
  interaction_probabilities ← sigmoid(FFN(aggregated_features))
  return interaction_probabilities

Function: EGRET(protein, window_size)
  condensed_features ← LocalFeatureExtractor(
    protein.features, window_size)
  aggregated_features ← EdgeAggregatedAttention(
      condensed_features, protein.edge_features, protein.neighbors)
  interaction_probabilities ← NodeLevelClassifier(aggregated_features)
  return interaction_probabilities
```

Algorithm 2 Pair-EGRET Architecture

```
Function: SiameseEGRETNetwork(receptor, ligand, window_size)
  condensed_features_r ← LocalFeatureExtractor(receptor.features, window_size)
  aggregated_features_r ← EdgeAggregatedAttention(
      condensed_features_r, receptor.edge_features, receptor.neighbors)
  condensed_features_l ← LocalFeatureExtractor(ligand.features, window_size)
  aggregated_features_l ← EdgeAggregatedAttention(
      condensed_features_l, ligand.edge_features, ligand.neighbors)
  return aggregated_features_r, aggregated_features_l

Function: PositionalEncoder(features)
  pos_encoding ← positional encoding following Equation 4
  return features + pos_encoding

Function: MultiHeadedCrossAttention(receptor_features, ligand_features, n)
  attention_heads_r, attention_heads_l ← n attention heads from receptor_features
    and ligand_features following Equation 5.
  attention_features_r ← layer normalize(receptor_features + concatenate(attention_heads_r))
  attention_features_l ← layer normalize(ligand_features + concatenate(attention_heads_l))
  return attention_features_r, attention_features_l

Function: PairwiseClassifier(receptor_features, ligand_features)
  initialize interactions
   for residue_r ∈ receptor do
      for residue_l ∈ ligand do
          interactions_rl ← sigmoid(FFN(concatenate(receptor_features, ligand_features)))
          interactions_lr ← sigmoid(FFN(concatenate(ligand_features, receptor_features)))
          interactions.append(average(interactions_rl, interactions_lr))
      end for
   end for
  return interactions

Function: InterfaceRegionClassifier(receptor_features, ligand_features)
  receptor_interface ← sigmoid(FFN(receptor_features))
  ligand_interface ← sigmoid(FFN(ligand_features))
  return receptor_interface, ligand_interface

Function: PairEGRET(receptor, ligand, window_size, dimension, n_heads)
  aggregated_features_r, aggregated_features_l ← SiameseEGRETNetwork(
      receptor, ligand, window_size)
  position_encoded_r ← PositionalEncoder(aggregated_features_r)
  position_encoded_l ← PositionalEncoder(aggregated_features_l)
  attention_features_r, attention_features_l ← MultiHeadedCrossAttention(
      position_encoded_r, position_encoded_l, n_heads)
  pairwise_interactions ← PairwiseClassifier(attention_features_r, attention_features_l)
  receptor_interface, ligand_interface ← InterfaceRegionClassifier(
      attention_features_r, attention_features_l)
  return pairwise_interactions, receptor_interface, ligand_interface
```

2 Supplementary Figures and Tables

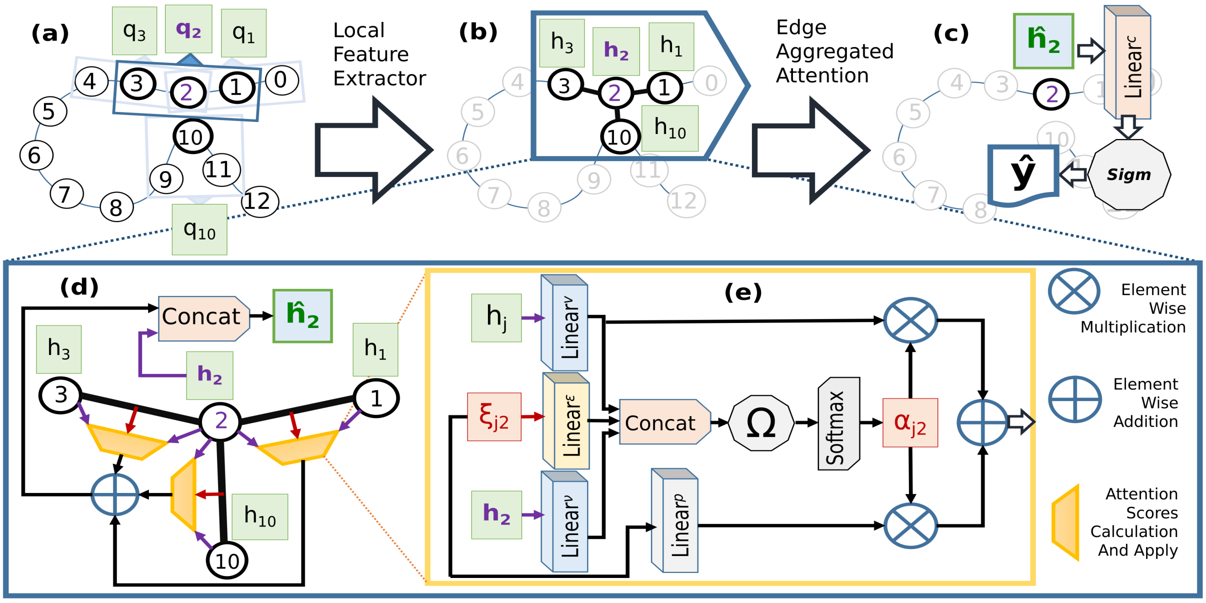

Figure S1: Schematic diagram of the overall pipeline of EGRET being applied to a dummy protein having 13 residues. **(a)** Local feature extractor (with window size w_local = 3). **(b)** Edge-aggregated graph attention layer applied to residue 2 with neighborhood $N_2 = \{1, 3, 10\}$. **(c)** Node level classifier applied to final representation $\hat{h_2}$ of node 2. **(d)** The details of the edge-aggregated graph attention layer in an expanded form. **(e)** The expanded form of the module that calculates the attention scores for aggregation. This figure has been taken from [Mahbub and Bayzid, 2022].

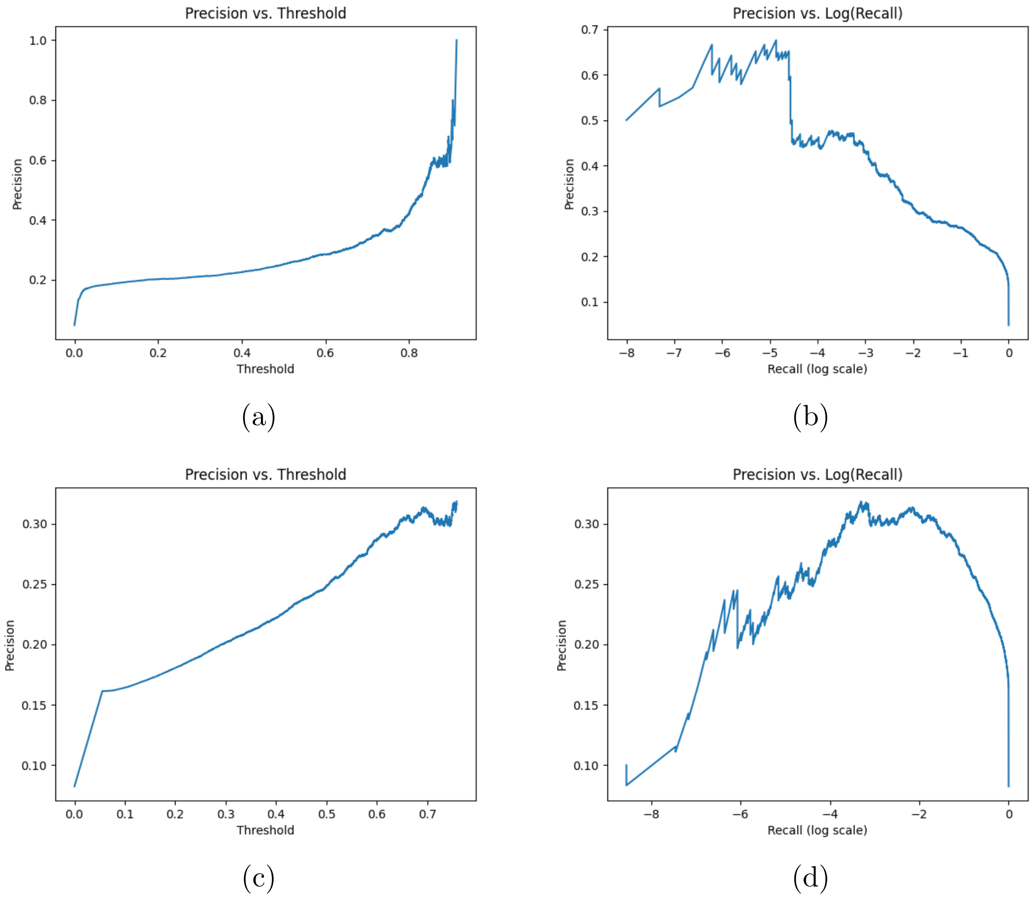

Figure S2: **(a-b)** Precision vs. Threshold and Precision vs. Recall (in log scale) plot for Pair-EGRET in the interface region prediction task on DBD5 test set, **(c-d)** Precision vs. Threshold and Precision vs. Recall (in log scale) plot for Pair-EGRET in the interface region prediction task on MASIF test set.

Table S1: Summary of the datasets used in this study. Positive samples represent the number of interacting residue pairs within the complexes. In contrast, negative samples account for the total number of non-interacting residue pairs within the same complexes.

Dataset	Samples	Train	Validation	Test	Total
DBD5	Complexes	140	35	55	230
	Positive samples	12,866 (9.09%)	3,138 (0.2%)	4,871 (0.1%)	20,875 (0.3%)
	Negative samples	128,660 (90.9%)	1,874,322 (99.8%)	4,953,446 (99.9%)	6,956,428 (99.7%)
Dockground	Complexes	236	60	100	396
	Positive samples	14,007 (9.09%)	3,940 (0.05%)	5,905 (0.04%)	23,852 (0.11%)
	Negative samples	140,070 (90.9%)	7,199,540 (99.94%)	12,673,885 (99.95%)	20,013,495 (99.88%)
MaSIF	Complexes	1890	470	787	3147
	Positive samples	308,441 (9.09 %)	77,903 (0.28%)	94,135 (0.26%)	480,479 (0.74%)
	Negative samples	3,084,229 (90.9%)	27,074,411 (99.71%)	34,833,241 (99.73%)	64,991,881 (99.26%)

Table S2: Set of working parameters for Pair-EGRET used in both pairwise PPIS prediction and interface region prediction tasks.

Hyperparameter	Value	Hyperparameter	Value
Protein language model	ProtBERT	Patience before stopping training	50
Optimizer	Adam	Batch size	32
Learning rate scheduler	StepLR	Maximum residue sequence length	1000
Learning rate	0.01	Neighborhood size for local features	21
Weight decay	0.0001	Number of GAT layers	2
Scheduler step size	60	Number of attention heads	4
Scheduler reduction rate	0.3	Attention layer hidden size	64
Total epochs	1000	Dropout rate	0.2

Table S3: Performace of Pair-EGRET on four subsets of DBD5 test dataset after excluding structurally similar proteins.

RMSD threshold	Number of excluded complexes	Median AUROC	AUPRC
0.25	0	0.888	0.0173
0.50	2	0.875	0.0162
0.75	4	0.872	0.0153
1.00	9	0.871	0.0150

Table S4: Precision scores of the top N high confidence predictions per complex by Pair-EGRET on pairwise interaction site prediction of DBD5 and Dockground test sets. *Min Confidence is the minimum probability score predicted by Pair-EGRET for these N residue pairs.

N	DBD5		Dockground	
	Precision	Min. Confidence*	Precision	Min. Confidence*
10	0.351	0.92	0.257	0.87
20	0.269	0.91	0.233	0.86
30	0.261	0.90	0.233	0.84
40	0.265	0.89	0.238	0.84
50	0.261	0.89	0.235	0.84
60	0.259	0.88	0.220	0.83
70	0.257	0.88	0.210	0.83
80	0.248	0.88	0.208	0.83
90	0.252	0.87	0.208	0.83
100	0.250	0.86	0.204	0.82

Table S5: Performance evaluation of Pair-EGRET and Alphafold-Multimer [Evans et al.] on Antibody-Antigen subset of the DBD5 test set. We also provide the deposit and release date of each complex. Please note that all the complexes were deposited and released before 2018, while Alphafold-Multimer was trained on all complexes up to 2018-04-30, with the current variant further fine-tuned on complexes until 2021-09-30. The higher scores are shown in bold font.

Complex	Deposit Date	Release Date	AUROC		AUPRC		F1		Precision		Recall	
(PDB ID)			Pair-EGRET	AFM	Pair-EGRET	AFM	Pair-EGRET	AFM	Pair-EGRET	AFM	Pair-EGRET	AFM
3RVW	2017-05-05	2017-05-24	**0.907**	0.549	0.004	**0.074**	0.009	**0.065**	0.004	**0.048**	**0.543**	0.100
4GXU	2012-09-04	2012-12-19	0.845	**0.925**	0.001	**0.823**	0.002	**0.822**	0.001	**0.795**	0.474	**0.851**
3V6Z	2011-12-20	2013-02-06	**0.965**	0.499	**0.036**	0.001	**0.059**	0.000	**0.030**	0.000	**0.778**	0.000
4FQI	2012-06-25	2012-08-22	**0.832**	0.542	0.005	**0.091**	0.006	**0.091**	0.003	**0.098**	**0.352**	0.085
4G6J	2012-07-19	2012-12-19	**0.970**	0.504	**0.065**	0.010	**0.062**	0.009	**0.033**	0.008	**0.568**	0.011
3HI6	2009-05-19	2009-09-22	**0.988**	0.521	**0.235**	0.035	**0.074**	0.032	**0.038**	0.025	**0.870**	0.043
3HMX	2009-05-29	2010-06-09	**0.959**	0.505	0.008	**0.012**	**0.016**	0.012	0.008	**0.014**	**0.621**	0.010
2W9E	2009-01-23	2009-02-03	**0.967**	0.505	**0.050**	0.013	**0.057**	0.012	**0.029**	0.011	**0.750**	0.013
4G6M	2012-07-19	2012-12-19	**0.953**	0.886	0.023	**0.794**	0.050	**0.793**	0.027	**0.816**	0.424	**0.772**
4DN4	2012-02-08	2012-10-03	**0.965**	0.899	0.032	**0.734**	0.074	**0.727**	0.039	**0.667**	**0.615**	0.800
3L5W	2009-12-22	2010-04-14	**0.967**	0.810	0.018	**0.679**	0.037	**0.674**	0.019	**0.738**	**0.640**	0.620
2VXT	2008-07-10	2009-06-23	**0.968**	0.886	0.032	**0.820**	0.058	**0.817**	0.031	**0.867**	0.364	**0.773**
3G6D	2009-02-06	2009-04-07	**0.958**	0.792	0.027	**0.646**	0.057	**0.640**	0.030	**0.706**	0.402	**0.585**
3EO1	2008-09-26	2008-12-02	**0.974**	0.563	0.027	**0.103**	0.052	**0.097**	0.027	**0.079**	**0.552**	0.126
3EOA	2008-09-26	2009-04-14	**0.960**	0.500	**0.022**	0.000	**0.034**	0.000	**0.018**	0.000	**0.388**	0.000
3MXW	2010-05-07	2010-05-26	**0.974**	0.589	0.035	**0.170**	0.047	**0.169**	0.024	**0.160**	**0.764**	0.180

Table S6: Precision, true positive rate (TPR) and false positive rate (FPR) scores of Pair-EGRET and NEA on predicting interface region of the complexes included in the case study.

Complex	Precision		TPR		FPR	
(PDB ID)	Pair-EGRET	NEA	Pair-EGRET	NEA	Pair-EGRET	NEA
3HI6	0.645	0.548	0.803	0.656	0.050	0.061
1JTD	0.522	0.479	0.667	0.625	0.095	0.106
3L89	0.195	0.156	0.419	0.581	0.212	0.385

Table S7: Median AUROC scores of Pair-EGRET for predicting pairwise interaction sites in DBD5 test complexes, evaluated with various combinations of node-level features.

Node features combination	Median AUROC (DBD5)	Improvement by feature
With both features	**0.88828**	-
Without physicochemical features	0.8031	+8.518%
Without ProtBERT-based features	0.7866	+10.168%

Table S8: Median AUROC scores of Pair-EGRET for predicting pairwise interaction sites in DBD5 test complexes, comparing the impact of different modules in the Pair-EGRET architecture.

Model architecture	Median AUROC (DBD5)	Improvement by module
Our full framework	**0.88828**	-
Without cross-attention	0.8632	+2.508%
Without positional encoder	0.84991	+3.837%

Table S9: Performance of models with simpler architectures compared to Pair-EGRET on PPIS prediction of DBD5 test complexes using the same set of enhanced features as Pair-EGRET.

Model architecture	Median AUROC (DBD5)
Siamese feed-forward neural network	0.6947
1D Convolutional neural network	0.7112
Attention-based network	0.7889
GAT with a single graph convolution layer	0.8232

References

J. Atwood and D. Towsley. Diffusion-convolutional neural networks. *Advances in neural information processing systems*, 29, 2016.

H. M. Berman, J. Westbrook, Z. Feng, G. Gilliland, T. N. Bhat, H. Weissig, I. N. Shindyalov, and P. E. Bourne. The protein data bank. *Nucleic acids research*, 28 (1):235–242, 2000.

T. Chen and C. Guestrin. Xgboost: A scalable tree boosting system. In *Proceedings of the 22nd acm sigkdd international conference on knowledge discovery and data mining*, pages 785–794, 2016.

B. Dai and C. Bailey-Kellogg. Protein interaction interface region prediction by geometric deep learning. *Bioinformatics*, 37(17):2580–2588, 2021.

J. Dunbar, K. Krawczyk, J. Leem, T. Baker, A. Fuchs, G. Georges, J. Shi, and C. M. Deane. Sabdab: the structural antibody database. *Nucleic acids research*, 42(D1): D1140–D1146, 2014.

D. K. Duvenaud, D. Maclaurin, J. Iparraguirre, R. Bombarell, T. Hirzel, A. Aspuru-Guzik, and R. P. Adams. Convolutional networks on graphs for learning molecular fingerprints. *Advances in neural information processing systems*, 28, 2015.

R. Evans, M. O'Neill, A. Pritzel, N. Antropova, A. Senior, T. Green, A. Žídek, R. Bates, S. Blackwell, J. Yim, et al. Protein complex prediction with alphafold-multimer. 2022; biorxiv doi: 10 march 2022, preprint: not peer reviewedhttps. *doi. org/10.1101/2021.10*, 4.

A. Fout, J. Byrd, B. Shariat, and A. Ben-Hur. Protein interface prediction using graph convolutional networks. *Advances in neural information processing systems*, 30, 2017.

P. Gainza, F. Sverrisson, F. Monti, E. Rodolà, D. Boscaini, M. M. Bronstein, and B. E. Correia. Deciphering interaction fingerprints from protein molecular surfaces using geometric deep learning. *Nature Methods*, 17:184–192, 2019.

H. Hwang, T. Vreven, J. Janin, and Z. Weng. Protein–protein docking benchmark version 4.0. *Proteins: Structure, Function, and Bioinformatics*, 78(15):3111–3114, 2010.

B. Jing, S. Eismann, P. Suriana, R. J. L. Townshend, and R. Dror. Learning from protein structure with geometric vector perceptrons. In *International Conference on Learning Representations*, 2021. URL `https://openreview.net/forum?id=1YLJDvSx6J4`.

T. N. Kipf and M. Welling. Semi-supervised classification with graph convolutional networks. In *5th International Conference on Learning Representations, ICLR 2017, Toulon, France, April 24-26, 2017, Conference Track Proceedings*. OpenReview.net, 2017.

S. Mahbub and M. S. Bayzid. Egret: edge aggregated graph attention networks and transfer learning improve protein–protein interaction site prediction. *Briefings in Bioinformatics*, 23(2):bbab578, 2022.

U. Ogmen, O. Keskin, A. S. Aytuna, R. Nussinov, and A. Gursoy. Prism: protein interactions by structural matching. *Nucleic acids research*, 33(suppl_2):W331–W336, 2005.

B. G. Pierce, K. Wiehe, H. Hwang, B.-H. Kim, T. Vreven, and Z. Weng. Zdock server: interactive docking prediction of protein–protein complexes and symmetric multimers. *Bioinformatics*, 30(12):1771–1773, 2014.

A. Porollo and J. Meller. Prediction-based fingerprints of protein–protein interactions. *Proteins: Structure, Function, and Bioinformatics*, 66(3):630–645, 2007.

C. R. Qi, H. Su, K. Mo, and L. J. Guibas. Pointnet: Deep learning on point sets for 3d classification and segmentation. In *Proceedings of the IEEE conference on computer vision and pattern recognition*, pages 652–660, 2017.

R. Sanchez-Garcia, C. O. S. Sorzano, J. M. Carazo, and J. Segura. Bipspi: a method for the prediction of partner-specific protein–protein interfaces. *Bioinformatics*, 35(3): 470–477, 2019.

R. Sanchez-Garcia, J. Macias, C. Sorzano, J. Carazo, and J. Segura. Bipspi+: Mining type-specific datasets of protein complexes to improve protein binding site prediction. *Journal of Molecular Biology*, 434(11):167556, 2022.

V. G. Satorras, E. Hoogeboom, and M. Welling. E (n) equivariant graph neural networks. In *International conference on machine learning*, pages 9323–9332. PMLR, 2021.

K. T. Schütt, F. Arbabzadah, S. Chmiela, K. R. Müller, and A. Tkatchenko. Quantum-chemical insights from deep tensor neural networks. *Nature communications*, 8(1): 13890, 2017.

R. Townshend, R. Bedi, P. Suriana, and R. Dror. End-to-end learning on 3d protein structure for interface prediction. *Advances in Neural Information Processing Systems*, 32, 2019.

P. Velickovic, G. Cucurull, A. Casanova, A. Romero, P. Lio, Y. Bengio, et al. Graph attention networks. 2018.

T. Vreven, I. H. Moal, A. Vangone, B. G. Pierce, P. L. Kastritis, M. Torchala, R. Chaleil, B. Jiménez-García, P. A. Bates, J. Fernandez-Recio, et al. Updates to the integrated protein–protein interaction benchmarks: docking benchmark version 5 and affinity benchmark version 2. *Journal of molecular biology*, 427(19):3031–3041, 2015.

R. Wang, X. Fang, Y. Lu, C.-Y. Yang, and S. Wang. The pdbbind database: methodologies and updates. *Journal of medicinal chemistry*, 48(12):4111–4119, 2005.
